# Genomic and environmental selection patterns in two distinct lettuce crop–wild hybrid crosses

**DOI:** 10.1111/eva.12043

**Published:** 2013-01-24

**Authors:** Yorike Hartman, Brigitte Uwimana, Danny A P Hooftman, Michael E Schranz, Clemens C M van de Wiel, Marinus J M Smulders, Richard G F Visser, Peter H van Tienderen

**Affiliations:** 1 Institute for Biodiversity and Ecosystem Dynamics, Universiteit van AmsterdamAmsterdam, The Netherlands; 2 Wageningen UR Plant Breeding, Wageningen University and Research CentreWageningen, The Netherlands; 3 Centre for Ecology and HydrologyWallingford, UK; 4 Biosystematics Group, Wageningen University and Research CentreWageningen, The Netherlands

**Keywords:** crop–wild hybrids, genetically modified crops, genotype by environment interaction, *Lactuca*, Quantitative Trait Loci, risk assessment

## Abstract

Genomic selection patterns and hybrid performance influence the chance that crop (trans)genes can spread to wild relatives. We measured fitness(-related) traits in two different field environments employing two different crop–wild crosses of lettuce. We performed quantitative trait loci (QTL) analyses and estimated the fitness distribution of early- and late-generation hybrids. We detected consistent results across field sites and crosses for a fitness QTL at linkage group 7, where a selective advantage was conferred by the wild allele. Two fitness QTL were detected on linkage group 5 and 6, which were unique to one of the crop–wild crosses. Average hybrid fitness was lower than the fitness of the wild parent, but several hybrid lineages outperformed the wild parent, especially in a novel habitat for the wild type. In early-generation hybrids, this may partly be due to heterosis effects, whereas in late-generation hybrids transgressive segregation played a major role. The study of genomic selection patterns can identify crop genomic regions under negative selection across multiple environments and cultivar–wild crosses that might be applicable in transgene mitigation strategies. At the same time, results were cultivar-specific, so that a case-by-case environmental risk assessment is still necessary, decreasing its general applicability.

## Introduction

The chance of crop alleles to introgress into their wild relatives is highly dependent on genetic and environmental selection patterns (Barton 2001; Stewart et al. 2003). For crop alleles to become permanently established in the wild population after single hybridization events, hybrid genotypes should confer a selective advantage in a particular environment (Burke and Arnold 2001; Rieseberg et al. 2007). Introgression of crop genes into a recipient population starts with F_1_ hybrids, with equal contributions of crop and wild genomes, genome-wide heterozygosity, and strong linkage disequilibrium (LD). In subsequent generations, a range of new genotypes is formed as a result of recombination and segregation in meiosis and the creation of new individuals by outcrossing or selfing. However, since the genetic background changes rapidly in the first phases of the introgression process, selection patterns may differ between early- and late-generation hybrids, as well as among individual plants within a certain category of hybrids (Barton 2001). Such patterns that affect the outcome of hybridization are not only interesting from a theoretical point of view (Rieseberg et al. 2000; Burke and Arnold 2001) but are also of high interest to Environmental Risk Assessment (ERA). Specifically, to what extent genomic selection patterns can be generalized across different cultivars and whether the performance of hybrids differs between early- and late-generations and different environments (EFSA 2011).

The performance of crop–wild hybrids can differ depending on the cultivar and wild parental lines used to produce specific crosses. In experiments employing crop–wild hybrids from several crosses with different parental lines, variation was found in life history and fitness traits, such as germination, seed production and survival between different crossing populations in oilseed rape (Hauser et al. 1998), sunflower (Mercer et al. 2006) and sorghum (Muraya et al. 2012). These differences in fitness response might also imply that selection acts on different regions in the genome. Recently, Quantitative Trait Loci (QTL) analysis on fitness characteristics measured in field trials has been used to study genomic selection patterns in crop–wild hybrids (Baack et al. 2008; Dechaine et al. 2009; Hartman et al. 2012), but little remains known of how differences in life history and fitness traits between different cultivar–wild-type crosses translate to differences in genomic selection patterns. With the production of high density integrated and consensus maps it becomes possible to compare QTL results between different cultivar–wild-type crosses (Hund et al. 2011; Swamy and Sarla 2011).

After a single hybridization event, several processes play a role: hitchhiking effects because of linkage drag, heterosis, epistasis and transgressive segregation interact to determine hybrid fitness (Stewart et al. 2003; Johansen-Morris and Latta 2006) and so influence the introgression chances of crop alleles. Epistasis is more thought to contribute to hybrid breakdown through the disruption of co-adapted gene complexes (Rieseberg et al. 2000), while heterosis and transgressive segregation can contribute to an increase in the performance of some hybrid lines relative to the wild parent (Burke and Arnold 2001). Hence, we focus on the latter processes in this study (but see Uwimana et al. (2012b) for a study on epistasis in lettuce) and we use two distinct hybrid generations: early generation backcross (BC) lines in which heterosis and transgression effects can occur and Recombinant Inbred Lines (RILs) with only transgressive effects.

Heterosis is most pronounced in early-generation hybrids, especially after hybridization between closely related species or inbred lines (Rieseberg et al. 2000), because of high levels of heterozygosity. Heterosis may be due to dominance (masking of deleterious alleles), overdominance (single-locus heterosis) and epistasis (enhanced performance of traits derived from different lineages due to non-additive interactions of QTL) effects (Rieseberg et al. 2000). It has been found many times in plants (Rhode and Cruzan 2005; Muraya et al. 2012), animals (Hedgecock et al. 1995) and insects (Bijlsma et al. 2010).

Transgressive phenotypes include hybrid plants that exceed the parental phenotype in a negative or a positive direction (Rieseberg et al. 2000). Transgressive phenotypes arise if parental species contain alleles with opposing effects, where some lines derive the positively contributing alleles from both parents and others derive the negatively contributing alleles, leading to hybrid genotypes that are more extreme than the parental lines (Lynch and Walsh 1998). In a review of 171 studies on segregating plant and animal hybrids, Rieseberg et al. (1999) showed that in 155 studies at least one transgressive trait was reported and that 44% of 1229 traits examined were transgressive. These studies show that both heterosis and transgressive segregation are widespread phenomena in hybridizing species (Rieseberg et al. 1999, 2003), suggesting that there is a high likelihood that at least some crop–wild hybrids have an increased fitness relative to the wild type in a given environment (Johansen-Morris and Latta 2006; Latta et al. 2007). Therefore, rather than estimating average hybrid fitness, it is necessary to view the entire fitness distribution of the hybrid lineages and identify how many individual hybrid lineages outperform the wild relative and when.

In addition to the potentially different response of hybrids from different parental lines, or from early- and late-generations, hybrid performance is also subject to Genotype × Environment (G × E) interactions (Barton 2001; Hails and Morley 2005). For example, several QTL studies that compared hybrid performance between greenhouse and field environments have shown that different traits and loci were favoured because of different selection pressures (Martin et al. 2006; Latta et al. 2007; Hartman et al. 2012). Similarly, hybrid fitness selection patterns differ across different natural environments (Weinig et al. 2003) and as a consequence of varying stresses, such as competition (Mercer et al. 2007). This suggests that hybrid fitness might be weakly correlated across divergent environments (Latta et al. 2007) and that as a result of these G × E interactions different hybrid lineages, and consequently alleles, might be selected for in different environments (Mercer et al. 2007). Moreover, hybridization between two wild parental species can lead to the colonization of new habitats previously unavailable to either of the parental species (Rieseberg et al. 2007). Therefore, the hybrid fitness distributions of different types of crosses and generations should also be considered in different environments, including the original wild habitat and novel environments, as we have done in this study.

In this study, we used progeny from different crosses between the crop lettuce (*Lactuca sativa* L.) and the wild-type prickly lettuce (*Lactuca serriola* L.). These species are fully cross-compatible and interfertile without any crossing barriers (Koopman et al. 2001). A recent study suggested that a substantial part of wild *L. serriola* plants in Europe (7%) show evidence of previous introgression of alleles from *L. sativa* (Uwimana et al. 2012a). In addition, it was demonstrated that compared with the wild parent up to four hybrid generations had higher average germination and survival rates in the field (Hooftman et al. 2005, 2007, 2009). Moreover, part of the crop genome was selectively advantageous leading to skewed crop–wild allele distributions (Hooftman et al. 2011). Although it is often assumed that crop alleles confer negative fitness effects in the wild habitat (Stewart et al. 2003), this suggests that in lettuce parts of the crop genomic background contribute to higher hybrid fitness and, therefore, potentially to the transfer of crop alleles to the wild population.

As different generations, early BC lines as well as late-generation RILs were used, originating from different parental lines. We employed these hybrid lineages and their parents in a location with sandy soil, which is similar to the natural habitat in which *L. serriola* occurs, and one with clay soil, which can be considered as a novel habitat given the current distribution of *L. serriola* (Hooftman et al. 2006). In a previous study, we identified two genomic regions under selection in the RILs, one where the crop genomic background was selectively beneficial and one where the wild genomic background was selectively beneficial (Hartman et al. 2012). In this study, we extend this analysis to the comparison with BC lines employed in the same experiment as the RILs and, in addition, studied the performance of individual hybrid lineages for both crossing types. This design allowed us to study similarities and differences in genomic selection patterns between different lettuce cultivar–wild crosses, hybrid performance in early- and late-generation hybrids and environmental influence on hybrid fitness distributions. We address these specific questions: (i) Which crop genomic regions are under positive or negative selection and are these similar or different between the BC and RIL crossing populations? (ii) Do the crop–wild hybrid populations differ in their fitness distribution and do they include hybrid lineages that perform better than the wild parent? (iii) Are there environment specific effects on the fitness distributions? In particular, is there an indication that introgression is more likely to occur in a novel habitat compared to the original habitat of the wild relative? Finally, we discuss the likelihood of crop gene transfer to the wild relative and the implications for ERA procedures.

## Material and methods

### Plant material

In this study, two different lettuce crop–wild crosses were employed. We used 98 lines of an existing RIL population (selfed for nine generations) derived from a cross between the cultivar *L. sativa* cv. Salinas (Crisphead) and Californian *L. serriola* (UC96US23; Johnson et al. 2000; Argyris et al. 2005; Zhang et al. 2007). In addition, we used 98 backcross lines selfed for one generation (BC_1_S_1_) from a cross between the cultivar *L. sativa* cv. Dynamite (Butterhead) and a *L. serriola* collected near the town of Eys, the Netherlands (designated cont83 in Van de Wiel et al. (2010); further referred to as *L. serriola* (Eys).

*Latuca sativa* was used as the pollen donor to mimic a hybridization event due to pollen flow from the crop to a neighbouring wild population. The F_1_ hybrid plant was subsequently backcrossed to the wild-type, creating a BC_1_ generation and each BC_1_ was then selfed to create a BC_1_S_1_ population. Crossing followed the protocols by (Nagata 1992) and (Ryder 1999), and is described in detail in Hooftman et al. (2005). Note that BC_1_ individuals were genotyped, whereas the BC_1_S_1_ were used in the experiments (see below).

Both wild *L. serriola* parents used in the crosses have leaves that are long and serrated, and contain a white latex substance. Plants develop up to 2 mm long spines on downside leaf midribs as well as on the base of the main stem. *Lactuca serriola* develops a rosette and flowers in July–August with many reproductive side shoots in the inflorescence and at the base of the plant. Capitula (flower heads) produce approximately 15–20 florets that develop into brown single-seeded achenes (further referred to as seeds). When seeds are ripe the involucral bracts become reflexed. *Lactuca serriola* occurs predominantly in ruderal sites, for example, along roads, railways and construction sites. This species is an annual that survives the winter mainly as seed, but also occasionally as small rosettes (Y. Hartman, field observation). Lettuce mainly reproduces by selfing, but research has shown that up to 5% outcrossing rates can be reached via insect pollination (D'Andrea et al. 2008; Giannino et al. 2008).

In contrast, the crop-types of *L. sativa* used in this study do not have spines and leaves are broad instead of serrated and do not contain latex. Plants develop a compact head instead of a rosette and do not have reproductive side shoots at the base of the stem. The cultivar group of Crisphead typically develops a very dense head (de Vries 1997) and develops brown seeds, whereas the Butterheads develop a relatively loose head and white seeds. Both cultivars have erect involucral bracts when seeds are ripe, most likely selected for to prevent seed shattering (de Vries 1997).

### Experimental set-up and analysis

This study was conducted in two contrasting field sites. The soil at the first site, located in Sijbekarspel (SB), the Netherlands (N52°42′, E04°58′), consisted of nutrient rich and water retaining clay similar to agricultural conditions. The second site, located in Wageningen (WG), the Netherlands (N51°59′, E05°39′), was similar to the wild habitat with dry, nutrient-poor and sandy soil. The weather conditions during the experiment were not different between the two sites (see Table S1).

For a detailed description of the experimental set-up see Hartman et al. (2012). In short, both sites consisted of 12 blocks, each with all 98 RILs, 98 BC_1_ families and the parental lines. Blocks contained 200 squares (40 × 40 cm) to which lines were randomly assigned, leading to a total of 4800 squares. We started the experiment with 30 seeds sown in each square and followed plants during the entire life cycle. Squares were thinned leaving one individual to reach the adult stage. This means that the data consisted of fitness estimates for all 4800 plants (i.e. including survival) and on average measurements on 4221 plants for different phenotypic traits.

Statistical and QTL analysis were performed on data of traits measured in the field. On the basis of the fitness QTLs found, we could distinguish ‘fitness QTL genotypes’ in both RILs and BCs, and compared their fitness distributions and the influence of the proportion crop genome.

### Traits measured

During the experiment, from May until October, we measured the following traits related to fitness (Table 1). Germination was measured 4 weeks after sowing and biomass measurements were done 7 weeks after sowing. Sites were visited daily to record the flowering date. At the seed set stage, the branches of the main inflorescence and basal reproductive side shoots were counted. In addition, we counted seeds from ten collected capitula and estimated the average number of seeds per capitulum. The number of shoots and branches was used to estimate the total number of capitula (See Hooftman et al. 2005 and Data S1). Subsequently, seed output was estimated by multiplying the average number of seeds per capitulum with the total number of capitula. We scored survival as a binary trait with 1 for survival until seed production and 0 for individuals that either died before seed set or did not complete their life cycle before the end of the growing season. We divided the number of seed-producing plants per line by twelve to calculate the survival rate. The final trait, seeds produced per seed sown (SPSS) was calculated using the following formula:


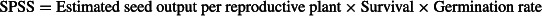
()

**Table 1:** Traits studied in a recombinant inbred lines (RILs) population of a *Lactuca sativa* cv. Salinas × *Lactuca serriola* (UC96US23) cross and in backcross (BC_1_S_1_) families of a *L. sativa* cv. Dynamite × *L. serriola* (Eys) cross

Plant stage	Trait	Abbreviation	Measurement and estimation method
Seedling	Germination rate	GM	Total number of seedlings divided by 30 seeds sown; seedlings counted 4 weeks after sowing; arcsine-square root transformation
Rosette	Biomass (g)	BM	Average dry weight of two rosettes; log transformation
Flowering	Days to first flower (day)	FLD	Number of days between sowing and the appearance of the first flower; log transformation
Seed set	Number of basal reproductive side shoots (count)	SHN	Number of basal reproductive side shoots which produced flowers, flower buds or seed heads; log transformation
	Number of branches main inflorescence (count)	BRN	Number of branches of the main inflorescence; log transformation
	Number of seeds per capitulum	SDC	Average number of seeds from ten collected capitula; no transformation necessary
	Total number of capitula	TC	Total number of capitula, estimation following Hooftman et al. (2005, eqn 1); log transformation
	Seed output	SDO	Total seed production, estimation following Hooftman et al. (2005); square root transformation
	Survival rate	SUR	Number of RIL or BC plants with seed production divided by twelve; arcsine-square root transformation
	Seeds produced per seed sown	SPSS	Number of seeds produced per seed sown, estimated by multiplying seed output, survival and germination rate; square root transformation

Of all traits, SPSS is the closest estimate of life cycle fitness and therefore referred to as the ‘main fitness trait’. The calculation of SPSS is slightly different than in Hartman et al. (2012), where we used average survival rate per line to calculate SPSS for each square, whereas here we used survival (e.g. either 0 or 1).

### Statistical analysis

We used PASW Statistics 17.0 (SPSS Inc 2009) for the statistical analyses. To improve normal distributions all traits were transformed, except for number of seeds per capitulum because this trait already had a normal distribution. Proportional data, such as survival and germination rates, were arcsine-square-root-transformed. Other traits were log-transformed (total number of capitula, number of branches, number of reproductive basal shoots and biomass) or square-root-transformed (SPSS and seed output). For each trait, the mean, standard deviation and heritability values were estimated. In addition, we also calculated the selection differentials for each trait by taking the covariance between the relative fitness and trait values (both with 12 data points per RIL or BC line). The relative fitness was calculated by dividing SPSS of each plant by the overall mean SPSS for a site.

We used heritability values to assess how much of the variation was due to genetic differences. Broad-sense heritability values (*H*^2^) were estimated as the proportion of the total variance accounted for by the genetic variance using the formula:


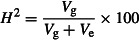


With *V*_g_ is the genetic variance and *V*_e_ is the environmental variance. *V*_g_ and *V*_e_ were inferred from between- and within-line variance components extracted with procedure VARCOMP (SPSS Inc 2009). Heritability values of family means (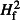) were estimated using the following formula (Chahal and Gosal 2002):


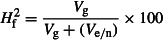


Where *n* is the average number of individuals per line measured for a certain trait (Table 2). The latter value indicates how well the family mean estimate resembles the true genetic value, given the number of replicates used, and is therefore important for the power of the QTL analyses.

**Table 2:** Estimated values of the mean and standard deviation, the broad-sense (*H*^2^) and family-mean (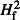) heritability, and the selection differentials for backcross (BC_1_S_1_) families, recombinant inbred lines and the parent lines

	RIL-parents						BC_1_S_1_-parents						RILs							BC_1_S_1_						
				
	*L. sativa* cv. Salinas			*L. serriola* (UC96US23)			*L. sativa* cv. Dynamite			*L. serriola* (Eys)								Selection differential							Selection differential	
																
Trait	Mean	SD	*n*	Mean	SD	*n*	Mean	SD	*n*	Mean	SD	*n*	Mean	SD	*n*	Broad sense *H*^2^ (%)	Family mean 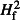 (%)	Absolute	Standar-dized	Mean	SD	*n*	Broad sense *H*^2^ (%)	Family mean 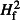 (%)	Absolute	Standar-dized
Sijbekarspel																										
GM (%)	60.8	18.9	12.0	25.3	11.0	12.0	62.2	13.4	12.0	48.3	17.3	12.0	52.8	14.6	12.0	27.9	82.3	4.6	0.316**	51.1	13.8	12.0	27.6	82.1	5.1	0.370**
BM (g)	1.083	0.586	12.0	0.542	0.334	11.0	1.011	0.384	12.0	0.762	0.315	12.0	0.681	0.335	11.9	14.1	66.2	0.037	0.112*	0.856	0.422	12.0	6.2	44.1	0.011	0.026
FLD (day)	115.0	–	1.0	93.8	3.1	12.0	127.4	8.2	5.0	115.2	14.4	10.0	94.3	4.8	10.2	89.5	98.9	−6.9	−1.454**	107.8	15.9	10.1	18.9	70.2	−9.1	−0.569**
SHN	–	–	0.0	4.2	2.1	12.0	–	–	0.0	10.3	3.3	6.0	3.3	1.9	9.9	50.5	91.0	1.4	0.717**	11.1	3.9	8.8	7.9	42.9	1.3	0.334**
BRN	–	–	0.0	36.3	8.0	12.0	–	–	0.0	31.0	5.5	6.0	28.3	5.2	10.2	14.1	62.7	4.1	0.793**	28.6	6.5	8.7	12.2	54.7	1.3	0.203**
SDC	–	–	0.0	10.0	3.4	12.0	–	–	0.0	13.5	3.3	6.0	7.2	2.4	10.0	75.8	96.9	6.8	2.773**	12.2	3.8	8.7	13.4	57.4	2.2	0.571**
TC	–	–	0.0	2566	492	12.0	–	–	0.0	3392	602	6.0	2011	421	10.2	62.0	94.3	462	1.097**	3411	746	8.7	12.3	54.9	296	0.397**
SDO	–	–	0.0	26 034	11 445	12.0	–	–	0.0	44 961	9588	6.0	14 411	6179	10.0	73.6	96.5	18 592	3.009**	41 888	16 703	8.6	7.7	41.8	11 057	0.662**
SUR (%)	0	–	12.0	100.0	0.0	12.0	0	–	12.0	50.0	52.2	12.0	56.9	14.6	12.0	76.4	97.5	43.2	2.960**	72.4	38.1	12.0	13.9	65.9	27.7	0.726**
SPSS	0	–	12.0	6921	4757	12.0	0	–	12.0	10 817	14 068	12.0	4700	2771	12.0	74.7	97.5			15 337	12 666	12.0	12.5	65.1		
Wageningen																										
GM (%)	72.8	21.2	12.0	35.0	14.1	12.0	82.2	10.3	12.0	66.1	14.1	12.0	66.4	18.0	12.0	24.4	79.5	7.9	0.437**	63.0	15.5	12.0	30.2	83.9	5.8	0.374**
BM (g)	1.543	0.818	12.0	0.955	0.593	12.0	1.336	0.453	12.0	0.991	0.515	12.0	1.183	0.554	11.9	16.5	70.2	0.076	0.137**	1.236	0.600	12.0	13.5	65.1	−0.004	−0.006
FLD (day)	104.0	–	1.0	82.1	3.3	12.0	122.5	7.8	4.0	103.4	11.4	12.0	91.4	4.5	10.7	89.5	98.9	−6.5	−1.445**	95.8	13.0	10.9	14.1	64.2	−5.5	−0.423**
SHN	–	–	0.0	1.3	0.9	12.0	1.0	–	1.0	10.1	2.4	11.0	2.4	1.3	10.1	54.6	92.4	1.2	0.935**	8.4	2.8	9.4	12.9	58.3	0.7	0.261**
BRN	–	–	0.0	41.8	8.2	12.0	25.0	–	1.0	28.8	9.4	11.0	29.1	5.5	10.1	16.5	66.6	4.8	0.869**	27.7	5.7	9.4	7.2	42.1	1.0	0.174**
SDC	–	–	0.0	19.1	3.1	12.0	12.7	–	1.0	17.9	1.7	11.0	12.6	2.6	9.6	64.0	94.5	3.3	1.251**	15.9	2.7	9.4	18.3	67.8	0.9	0.338**
TC	–	–	0.0	2333	433	12.0	4	–	1.0	3239	687	11.0	1887	351	10.1	74.9	96.8	453	1.289**	2888	573	9.4	13.9	60.2	179	0.312**
SDO	–	–	0.0	45 171	11 869	12.0	52	–	1.0	57 892	13 251	11.0	23 631	6865	9.7	68.1	95.4	13 866	2.020**	45 947	12 436	9.4	15.0	62.5	5557	0.447**
SUR (%)	0.0	–	12.0	100.0	0.0	12.0	8.3	28.9	12.0	91.7	28.9	12.0	57.1	12.9	12.0	80.0	98.0	43.0	3.325**	80.1	32.0	12.0	13.8	65.7	19.9	0.623**
SPSS	0	–	12.0	15 743	7638	12.0	3.6	12.5	12.0	35 027	15 497	12.0	9132	4984	12.0	73.9	97.4			22 688	14 647	12.0	13.3	66.7		

RIL, Recombinant Inbred Lines; *n*, average number of individuals per line or family; SPSS, seeds produced per seed sown. Abbreviations are listed in Table 1.

* Significant at 0.05 level.

** Significant at 0.01 level.

### Quantitative trait loci analysis

For RILs, the genetic map employed consisted of 1513 predominantly AFLP and EST derived SNP markers (http://cgpdb.ucdavis.edu/GeneticMapViewer/display/; map version: RIL_MAR_2007_ratio; Johnson et al. 2000; Argyris et al. 2005; Zhang et al. 2007); both map and markers were developed by the Compositae Genome Project website (http://compgenomics.ucdavis.edu).

**Figure 1:**
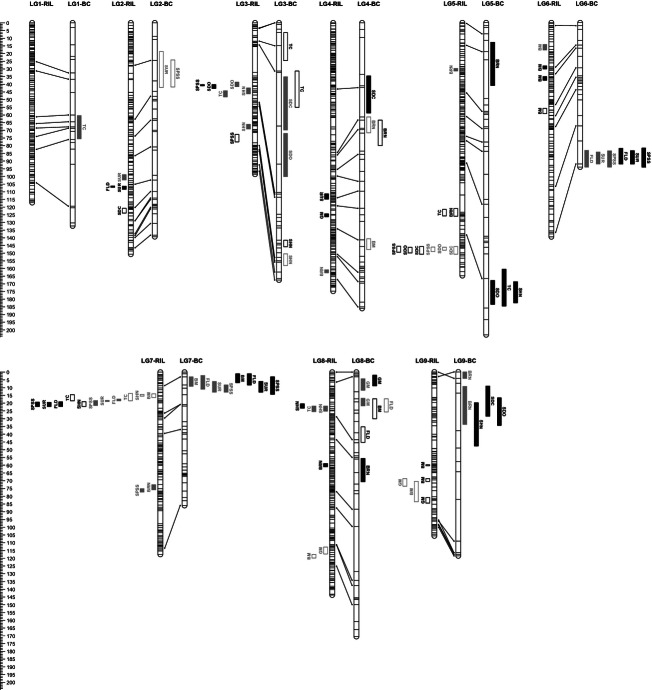
Positions of quantitative trait loci (QTL) in backcross (BC_1_S_1_) families of a *Lactuca sativa* cv. Dynamite × *L. serriola* (Eys) cross and a recombinant inbred lines (RIL) population of a *L. sativa* cv. Salinas × *L. serriola* (UC96US23) cross using composite interval mapping. Map distances (cm) are located on the left side. The same linkage groups of RIL and BC map are shown next to each other; markers are shown as horizontal lines. Linkage group names are shown at the top and dotted lines between linkage group bars indicate similar markers. RIL QTL are shown on the left side of linkage groups by black or grey bars, whereas BC QTL are shown on the right. Black bars indicate Wageningen QTL and grey bars indicate Sijbekarspel QTL. When the crop genomic background (*L. sativa*) gives a selective advantage (derived from the selection differentials shown in Table 2) the QTL is shown as an open bar; when the wild genomic background (*L. serriola*) gives a selective advantage the QTL is shown as a filled bar. The length of QTL bars is determined by the one-LOD confidence interval. Abbreviations are listed in Table 1.

For BC lines, the genetic map consisted of 347 SNP markers distributed over nine linkage groups (described in detail in Uwimana et al. 2012b). These were selected from 1083 SNPs, developed by the Compositae Genome Project (http://compgenomics.ucdavis.edu/compositae_SNP.php) from disease resistance and developmental genes in lettuce, using a customized Illumina GoldenGate array with markers polymorphic between the parent lines. Note that BC_1_ plants were genotyped and that their offspring (BC_1_S_1_) was used in the experiments. We conducted the QTL analyses in QTL Cartographer (version 2.5.008, Wang et al. 2010). RIL and BC_1_S_1_ data were analysed separately. We used Composite Interval Mapping (CIM) testing at 2 cm intervals and a stepwise regression method (forward and backward) with five background cofactors and a 10 cm window. Permutation tests were used to estimate a significance threshold of α = 0.05 for QTL using 1000 iterations (Doerge and Churchill 1996). Additive effects and one-LOD support intervals were obtained from the CIM results. MapChart 2.2 was used to draw the linkage map and QTL results (Voorrips 2002). The marker order of LG1, 3, 4, 7 and 8 of the BC map was reversed to be able to compare RIL and BC QTL; 80 markers were similar between the RIL and BC map (Fig. 1).

### Fitness distributions

To visualize variation in fitness for both sites, we ranked all 98 BC or RIL and parental lines based on the estimated average SPSS and plotted the estimated average SPSS of lines against their rank. In addition, we visualized the influence of major fitness QTL on the fitness distributions. We focussed specifically on the genomic regions where BC and RIL fitness QTL co-localized across sites. Lines that we could unequivocally assign to a certain ‘fitness QTL genotype’ were colour-coded. Coloured lines had no missing data and all flanking markers were of one parental background. Colour-codes indicated if fitness QTL contained alleles from the crop or the wild parent or a combination of both parental lines. We also estimated the average rank per fitness QTL genotype indicating if a certain fitness QTL genotype had an average high or low rank.

### Influence of the proportion crop genome

To visualize the influence of the amount of crop genome on fitness, we plotted the estimated average SPSS of BC_1_ families and RILs against an estimate of the percentage of crop genome. This estimate was based on counting markers as coming from the crop or wild relative (missing data were excluded). The analysis was done for both sites and crossing types separately and included all 98 RIL or BC_1_ families and all parental lines.

First, we used a univariate linear regression to estimate the overall relationship between SPSS and the percentage of crop genome in R (version 2.14.0; R Development Core Team 2011). Second, we repeated this analysis, while excluding the effect of the two major fitness QTL by adding these as covariates (based on the genotype data that were also used for the fitness distributions), therefore estimating the relationship between the residual variation in SPSS and the percentage of crop genome. In this second analysis, we omitted genotypes for which the presence of the fitness QTL was ambiguous, either due to missing markers or a recombination event in the QTL interval. In addition, we estimated the average amount of crop genome per fitness QTL genotype.

## Results

### General survival

Survival of plants was comparable between sites. For RILs, 57.1% of plants survived until reproduction at WG and 56.9% survived at SB (Hartman et al. 2012). A higher percentage of BC individuals survived until reproduction at both sites; 80.1% for WG and 72.4% for SB.

### Parental lines

The main difference between the cultivars and wild parental lines is that most crop individuals died before seed production, whereas the majority of wild-type individuals survived and produced seeds (Table 2). In both SB and WG, only one *L. sativa* cv. Salinas individual survived until flower production, but died before reproductive characters could be recorded. Similarly, only one *L. sativa* cv. Dynamite individual survived until flower production in SB; in WG, four individuals survived until flowering but only one of them produced seeds in four capitula. Other trends are that crop cultivars had higher germination rates, higher biomass production and flowered later compared with the wild parental lines of the same cross (Table 2). In addition, all parental lines developed faster and flowered earlier in WG compared to SB.

### Heritability values and selection differentials

Heritability values patterns were more variable among BC lines than among the RILs, consistent with the larger genetic variation within and among these lines. For BC lines, biomass, number of reproductive basal shoots and seed output had the lowest heritability values in SB, whereas in WG, number of reproductive basal shoots and branch number had the lowest heritability values. At both sites, germination showed the highest broad-sense and family-mean heritability. For RILs, branch number, biomass and germination rate showed the lowest broad-sense and family-mean heritability values, whereas days until first flower showed the highest values at both sites.

For BC lines, broad-sense heritability values varied from 6.2% to 30.2% and family-mean heritability values varied from 41.8% to 83.9%. For RILs, these varied between 14.1% and 89.5% and 62.7% and 98.9%, for broad-sense and family-mean heritabilities respectively (Table 2), indicating that the replication level was adequate, given the environmental variation under field conditions.

The majority of selection differentials showed significant trends (Table 2), except for BC_1_S_1_ biomass in SB and WG. Across sites and crosses, all selection differentials indicated that higher values were favoured, with the exception of days to first flower. For this trait lower values were favoured, namely 6–7 days earlier flowering for RILs and 5–9 days for BC_1_ families.

### Quantitative trait loci analysis

For the BC_1_ families, we detected a total of 43 QTL for ten fitness and fitness-related traits distributed over all nine linkage groups (Table 3; Fig. 1). The Phenotypic Variation Explained (PVE) ranged from 6.4% to 42.8%. One to three QTL were detected per trait (mean 2.2) and 1-LOD support intervals varied between 4.2 and 34.7 cm (mean 13.7 cm). When the two field sites are combined for all ten traits, nine QTL were detected at both sites; the remaining 25 QTL were unique for one of the sites. QTL results of the RIL population are summarized in Fig. 1 and are described in more detail in Hartman et al. (2012, see Table S2). In short, a total of 49 QTL was detected and when the two field sites are combined, eleven QTL were found at both sites, whereas 27 QTL were unique for one of the sites.

**Table 3:** Positions of quantitative trait loci (QTL) in backcross (BC_1_S_1_) families of a *Lactuca sativa* cv. Dynamite × *Lactuca serriola* (Eys) cross using composite interval mapping. Quantitative trait loci results of the recombinant inbred lines population from a *L. sativa* cv. Salinas × *L. serriola* (UC96US23) cross are described in detail in Hartman et al. (2012; but see Table S2 for SPSS QTL). A positive additive effect indicates that crop genomic background (*L. sativa*) causes higher trait values, whereas a negative additive effect indicates that the wild genomic background (*L. serriola*) causes higher values. QTL on the same line have peak values within 5 cm

		Sijbekarspel					Wageningen				
			
LG	Trait	Position	One-LOD interval	Additive effect	PVE (%)	LOD	Position	One-LOD interval	Additive effect	PVE (%)	LOD
1	TC	70.7	60.4–75.5	−0.04	11.8	3.2					
2	SUR	30.5	18.7–42.1	0.15	6.4	3.0					
	SPSS	32.5	24.1–41.8	20.92	9.4	3.0					
3	TC						19.4	6.4–24.6	0.04	13.3	3.9
	TC						35.2	31.4–55.0	0.03	10.9	3.2
	SDC	51.2	35.1–69.8	−1.93	20.2	4.7					
	SDO	84.0	72.0–100.2	−19.63	19.1	4.5					
	SHN						144.6	141.6–145.8	0.07	12.8	4.2
	SHN	155.9	150.4–158.1	0.21	19.5	4.9					
4	SDC						46.2	34.6–58.7	−1.19	12.7	4.0
	BRN	63.3	61.3–71.6	0.04	10.4	3.5	68.6	63.3–80.0	0.03	8.5	2.9
	BM	141.3	140.6–147.7	−0.02	10.1	3.6					
5	BRN						27.8	12.8–41.2	−0.03	10.6	3.1
	TC						175.2	161.8–185.9	−0.04	16.1	3.6
	SDO						177.2	169.2–184.8	−14.97	18.7	4.7
	SHN						177.2	170.0–183.8	−0.11	31.2	7.6
6	SUR	91.6	84.9–92.7	−0.36	37.7	12.7	89.6	84.0–92.7	−0.39	42.8	14.2
	SPSS	92.7	84.1–94.7	−26.80	16.3	5.7	92.7	82.3–94.7	−30.54	18.4	5.9
	FLD	92.7	83.8–94.7	0.03	13.0	4.5	89.6	82.5–92.7	0.03	27.3	8.0
7	BM	6.3	3.1–9.1	0.04	24.3	7.5	3.1	1.1–6.8	0.06	24.5	7.5
	FLD	6.3	2.2–11.0	0.03	19.0	6.0	3.1	1.1–8.3	0.02	8.8	3.1
	SPSS	10.4	8.3–12.9	−30.58	20.6	6.9	10.4	3.1–14.3	−20.51	8.5	3.0
	SUR	10.4	6.0–12.9	−0.30	26.0	9.7	10.4	6.0–12.9	−0.31	26.5	10.2
8	GM	4.6	4.3–11.7	−0.09	17.8	4.5	3.1	2.0–8.8	−0.09	10.0	2.7
	GM	19.2	16.9–21.8	−0.10	22.4	6.1					
	FLD	19.2	17.2–25.7	−0.03	14.8	5.0					
	BM						24.5	17.2–30.0	−0.04	11.8	4.8
	FLD						41.5	35.3–45.3	−0.02	11.9	4.0
	BRN						62.7	55.7–70.6	−0.03	13.9	4.4
9	BRN	0.00	0.0–4.2	−0.04	10.9	3.2					
	SDC						15.9	9.3–28.8	−1.44	17.9	5.8
	BRN	19.9	9.4–34.1	−0.06	19.7	5.3					
	SDO						25.9	16.9–34.8	−17.79	26.5	6.5
	SHN						33.9	20.1–48.2	−0.06	11.8	3.2

PVE, Percentage Variation Explained; SPSS, seeds produced per seed sown; Abbreviations are listed in Table 1.

The comparison between RIL and BC QTL fitness clusters shows similarities but also differences (Fig. 1). For both crosses, there were two genomic regions where several QTL clustered including QTL for SPSS, the main fitness QTL. For the BC_1_, these regions were located at LG6 (bottom) and at LG7 (top, Fig. 1). The same QTL are found for SB and WG at these genomic locations and in both cases selection differentials indicated that the selective advantage was conferred by the wild allele for these QTL. At LG6 and LG7, the wild genomic background increased SPSS and survival rate and reduced days until first flower. At LG7, additional QTL were detected for biomass and again a selective advantage was conferred by the wild genomic background, increasing biomass.

For the RILs, a fitness cluster was found across sites at the bottom of LG5, whereas a second fitness cluster was situated at LG7 (Hartman et al. 2012), overlapping the cluster found for the BC population. At LG5, QTL for seeds per seed sown, seed output and seeds per capitulum were detected and a selective advantage was conferred by the crop allele (Fig. 1). This region corresponded with BC QTL found for seed output, shoot number and total capitula, but in contrast to the RIL QTL, no seeds per seed sown QTL was found and here the selective advantage was conferred by the wild rather than the crop allele. At LG7 and similar to BC results, a selective advantage was conferred by the wild allele QTL for SPSS, survival rate until seed set, and days to first flower, indicating that both crop varieties contained gene(s) for delayed reproduction. Additional RIL QTL found were total capitula, shoot number and biomass, and for these traits a selective advantage was conferred by the crop allele.

### Fitness distributions

**Figure 2:**
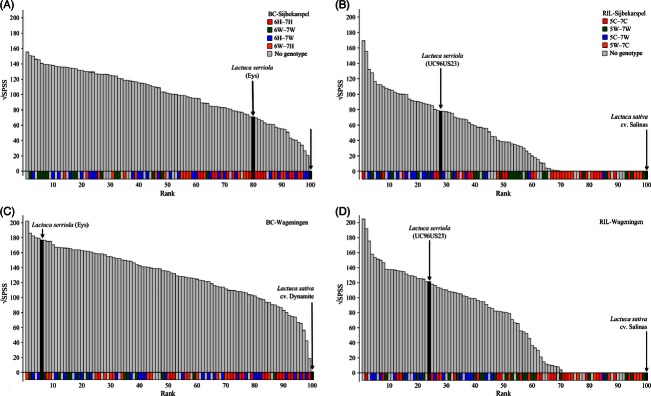
Fitness distributions across lines for (A) backcross (BC_1_S_1_) families in Sijbekarspel (SB), (B) recombinant inbred lines (RILs) in SB, (C) BC_1_S_1_ families in Wageningen (WG) and (D) RILs in WG. Each bar represents one line. Lines are ranked based on the average Seeds Produced per Seed Sown. Coloured squares below the *x*-axis indicate the genotype for genomic fitness regions on LG6 and 7 for BC lines, and LG5 and 7 for RILs; for genotype notation, see Table 4. Black squares indicate parent lines and grey squares indicate lines for which the genotype remains unknown.

Fitness distributions of RIL and BC crossing populations differed considerably. All BC lines had some seed output, whereas approximately 30% of RILs produced no seeds in SB and WG (Fig. 2). They either died before seed set or did not complete their life cycle before the end of the growing season. For RILs, the proportion of lines that performed better than the wild parent was comparable across sites, with 27% in SB and 23% in WG. For BC lines there was a considerable difference, with 79% of lines performing better than the wild parent in SB, whereas only 5% performed better in WG.

Given the QTL fitness regions, BC lines with a wild genomic background for LG6 and 7 (6W–7W) were expected to have the highest seed yield, whereas the opposite combination (6H–7H; H indicating that BC_1_ genotypes were heterozygous for these loci) should have the lowest seed yields. The 6W–7W lines (green bars) are indeed situated at the high-end of the fitness distributions, whereas the 6H–7H lines (red bars) are situated at the low-end side (Fig. 2). This is reflected in the average ranks of 24.0 out of 100 in SB and 30.5 in WG for 6W–7W lines, and 78.6 in SB and 77.9 in WG for 6H–7H lines (Table 4).

**Table 4:** Average rank and amount of crop genome of four genotypes (based on QTL of the main fitness trait seeds per seed sown) across 98 recombinant inbred lines (RILs) or backcross (BC_1_S_1_) families

	Average rank			
			
Genotype	Sijbekarspel	Wageningen	% crop genome	No. of lines
BC_1_S_1_ families				
6H–7H	78.6	77.9	31.0	16
6W–7W	24.0	30.5	21.0	13
6H–7W	51.9	52.7	25.1	27
6W–7H	56.9	46.9	25.8	20
No genotype	34.6	42.7	25.4	22
RILs				
5C–7C	52.9	51.7	52.1	21
5W–7W	51.3	53.1	51.0	23
5C–7W	27.6	28.9	50.2	16
5W–7C	76.5	73.1	52.0	13
No genotype	47.8	48.2	49.8	25

C, homozygous crop allele; W, homozygous wild allele; H, heterozygous crop and wild allele; QLT, quantitative trait loci.

For RILs, letters indicate genomic fitness regions on LG5 and 7 and for BC lines, letters indicate genomic fitness regions on LG6 and 7. For example, 5C–7C indicates crop genotype for the identified QTL on both LG5 and LG7; lines without sufficient information are joined into ‘No genotype’. No. of lines = number of BC or RIL lines in each category (each line with 12 replicates per site). % crop genome = average% of markers derived from the crop parent (BC_1_ or RIL).

Recombinant Inbred Lines with the crop genomic background for LG5 and the wild parental background for LG7 (5C–7W) were expected to have the highest fitness. Lines with this fitness QTL genotype (blue bars) are indeed mostly located at the high-end of the fitness distribution (Fig. 2) and had the highest average rank at both sites (27.6 of 100 in SB and 28.9 in WG, Table 4). RILs with the opposite combination, 5W–7C (orange bars), mainly situated at the low-end of the fitness distribution and had the lowest average rank of 76.5 in SB and 73.1 in WG.

These QTL fitness regions do not explain all variation of the fitness distributions as seen by the mixed distribution of the coloured bars (Fig. 2). The PVE of the QTL for seed production (SPSS) reflects the unexplained variation. The combined PVE for BC fitness QTL was approximately 27% (WG) to 37% (SB), and for RIL fitness QTL approximately 30% at both sites, implying that part of the variation went undetected.

### Influence of the proportion crop genome

**Figure 3:**
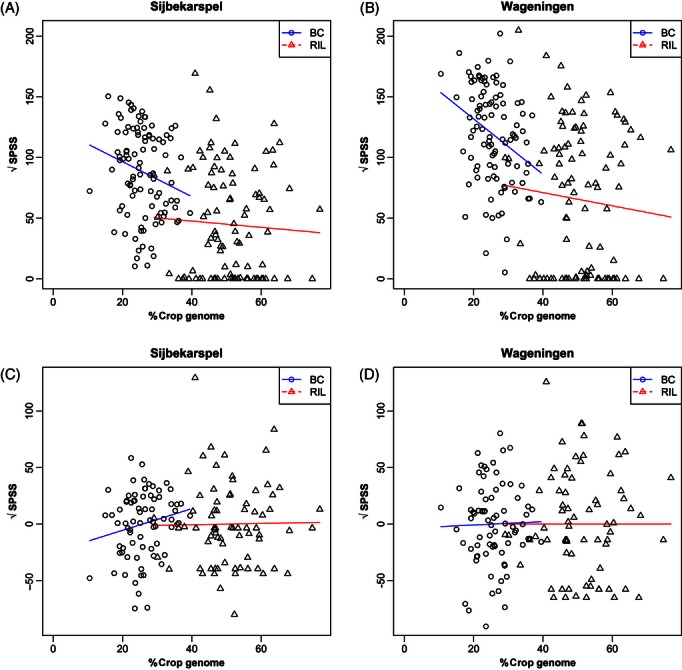
Relationship between the amount of crop genome (%) on the average Seeds Produced per Seed Sown (square-root-transformed) for each backcross (BC_1_) family and recombinant inbred line (RIL). (A, B) simple regression of fitness on crop genome%, and (C, D) residual regression after the effects of the two major fitness quantitative trait loci were taken out, as covariates; Sites: Sijbekarspel (A and C) and Wageningen (B and D). Dots indicate BC lines and triangles indicate RIL averages. Regression equations: 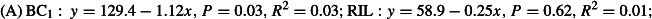 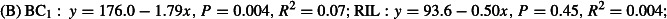 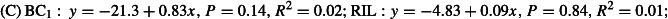 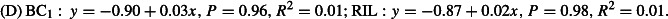

The average amount of crop genome was 23.7% for the BC_1_ lines, ranging from 10.5% to 39.5% (Fig. 3). For RILs, the average was 50.9%, ranging from 29.1% to 76.9%. There was a large spread in SPSS for both BC_1_S_1_ families and RILs that had approximately the same amount of crop genome (Fig. 3A,B). Consequently, for BC_1_S_1_ families only 3% (SB) to 7% (WG) was explained by the univariate linear regressions. *P*-values were significant (SB: *R*^2^ = 0.03, *P* < 0.05, df = 96; WG: *R*^2^ = 0.07, *P* < 0.01, df = 96). The estimated slopes of the linear regression were quite steep, with an increase in crop genome from 20% to 30% predicted to result in a reduction of 2271 seeds and 4699 seeds for SB and WG respectively (based on regression equations). For RILs, the explained variance was very low with 1.0% in SB and 0.4% in WG, and *P*-values were not significant (SB: *R*^2^ = 0.01, *P* = 0.62, df = 96; WG: *R*^2^ = 0.004, *P* = 0.45, df = 96).

The results of the regression analysis changed considerably for BC_1_ families when the variation in SPSS due to the two major fitness QTL was removed (Fig. 3C,D). The variation in SPSS explained by the linear regressions was lower and *P*-values were no longer significant (SB: *R*^2^ = 0.02, *P* = 0.14, df = 74; WG: *R*^2^ = 0.01, *P* = 0.96, df = 74). For RILs, the explained variance was even lower and non-significant.

For RILs, the average amount of crop genome was similar across fitness QTL genotypes (Table 4: 49.8–52.1%). The most advantageous BC_1_ fitness QTL genotype (6W–7W) had the lowest amount of crop genome (21.0%), whereas the least advantageous BC_1_ fitness QTL genotype (6H–7H) had the highest (31.0%), indicating that selection in this BC_1_ population might lead to a considerable purging of crop genes at these genomic locations.

## Discussion

### Overlapping and separate genomic regions are under selection

Quantitative trait loci results under field conditions may vary from site to site and genetic material used (Mercer et al. 2006; Muraya et al. 2012). In our case, the crop cultivar, as well as the wild parent, differed between the BC and RIL crossing population. Given this context, it is perhaps surprising that we found several key genomic regions affecting fitness traits in both crossings and environments, next to a number of substantial differences.

Both the BC and RIL populations had two genomic regions, one co-localized and one specific for each cross, with fitness QTL that were consistent across field sites. Fitness distributions and the average rank of fitness QTL genotypes (based on fitness QTL) confirmed that these genomic regions indeed had a substantial impact on the fitness of BC and RIL hybrid lineages. The majority of lines with the most selectively advantageous fitness QTL genotype displayed relatively high seed yields and averaged these groups showed the highest rank compared with other combinations of parental alleles. This pattern with few genomic regions of major impact is similar to QTL selection patterns found in slender wild oat (Latta et al. 2010) and in sunflower (Baack et al. 2008; Dechaine et al. 2009).

Seeds produced per seed sown QTL co-localized at the top of linkage group (LG) 7 for both BC and RILs. The selection differentials showed that the selective advantage was conferred by the wild allele, by favouring a higher SPSS, early flowering and higher survival rates. This QTL region is probably the result of the presence of a major gene for flowering, in which the crop allele confers a selective disadvantage by delaying bolting (Hartman et al. 2012). The second genomic region under selection was specific for each cross, with BC fitness QTL on the bottom of LG6 and RIL fitness QTL on the bottom of LG5. For BC QTL at LG6, it was again the wild allele that gave the selective advantage favouring earlier flowering, higher survival rates, and higher SPSS. These did not co-localize with any RIL QTL. In contrast, for the RIL QTL cluster of LG5, it was the crop allele that favoured SPSS, seed output and seeds per capitulum (Hartman et al. 2012).

### Genetic basis of better performing lines

At both field sites and for BC, as well as RIL crossing populations, there was a substantial number of hybrid lines that outperformed their respective wild parent, although hybrids on average produced less seeds per seed sown than the wild parent, with the exception of BC hybrids on clay soil that performed better than the wild parent (see below). This observed hybrid vigour concurs with the transgressive segregation observed in greenhouse experiments employing the same BC and RILs hybrid lineages, in which individual lines had an increased vigour under drought, nutrient limitation and salt stress (Hartman 2012; Uwimana et al. 2012b).

Heterosis, increased hybrid vigour in early-generation hybrids (Rieseberg et al. 2000; Johansen-Morris and Latta 2006), probably explains, for the larger part, that all BC_1_S_1_ families produced at least some seeds, even though these hybrids where backcrossed once to one of the parents. In contrast, approximately 30% of RILs produced no seed output. With each subsequent generation, heterozygosity rapidly decreases in a selfing species. Hence, a lettuce RIL population selfed for nine generations lines are virtually entirely homozygous and heterosis effects cannot account for the better performing lines in later generations (Burke and Arnold 2001). However, the higher fitness of early-generation lettuce hybrids may favour survival of hybrids with novel genotypes, thereby increasing the chances for these beneficial novel genotypes to be fixed in later generations (Johansen-Morris and Latta 2006; Latta et al. 2007).

The steep decline in fitness of BC_1_ families with a higher amount of crop genome indicates there might be a strong selection against and hence, a rapid elimination of crop genome in the first hybrid generations. This could be due to hitchhiking effects, since in early-generation hybrids many crop genes are in LD with genes under selection, as indicated by the lower amount of crop genome of the most advantageous BC_1_ fitness QTL genotype (based on fitness QTL). In contrast, LD is greatly reduced in 9^th^ generation RILs (Flint-Garcia et al. 2003; Stewart et al. 2003). Moreover, a positively selected crop gene was also segregating in the RIL population. In RILs, all genotypes have approximately the same amount of crop genome. This suggests that in later generations particular combinations of genes became important, independent of linkage drag, giving rise to transgressive segregation (Rieseberg et al. 1999, 2003).

Quantitative trait loci studies have consistently pointed at the additive effects of complementary genes of the two parental species as the most likely underlying genetic basis for transgressive segregation (Rieseberg et al. 1999, 2000; Burke and Arnold 2001). After hybridization, QTL with effects in opposing directions within each parent may recombine in the hybrids, resulting in some lettuce hybrids having a majority of QTL with positive effects leading to a high fitness, or with negative effects leading to a low fitness (Lynch and Walsh 1998; Rieseberg et al. 2007). Indeed, six to seven (BC and RILs results respectively) of the ten traits measured in this study show QTL with opposing effects, where in some genomic locations the crop parental allele is selectively advantageous and in other locations it is the wild parental allele.

Heterosis, linkage and transgressive segregation are not the only genetic processes underlying hybrid fitness. For example, Uwimana et al. (2012b) found epistasis effects in BC_1_ and BC_2_ generation lettuce hybrids when subjecting these to several stress treatments in greenhouse conditions. In later generations, these epistasis effects are more likely to contribute to the breakdown of co-adapted gene complexes (Rieseberg et al. 2000; Burke and Arnold 2001) and therefore lower hybrid fitness. This may also partly explain the 30% of RILs without any seed output.

Our results are based on two *L. serriola* genotypes, a European and an American accession. Genetic diversity in *L. serriola* is considerable (Van de Wiel et al. 2010), so it would be desirable to study more wild genotypes, for instance, as diallel combinations with crop varieties in future studies.

### Higher chance of introgression in novel habitats

Fitness distributions were different among the two habitats used, indicating that introgression of crop alleles through hybridization might be more likely to occur in novel habitats, as opposed to the natural wild habitat of the wild parent. More hybrid lineages performed better than *L. serriola* in the novel clay soil habitat than in the original sandy soil habitat (habitat requirement as described in Hooftman et al. (2006)), especially BC hybrid lineages. In spite of the fact that the selective advantage for the two BC fitness QTL was conferred by the wild allele, 79% of families performed better than the wild parent (*L. serriola* Eys) in clay soil, whereas only 5% of BC_1_S_1_ families performed better in sandy soil. The lower performance of the wild parent in the clay site was caused by a lower survival until reproduction, as well as a lower than average seed yield of reproducing plants. In addition, the PVE by fitness QTL (in total 36.9% in clay soil and 26.9% in sandy soil) indicates that not all fitness variation was explained by these fitness QTL and that apparently the increased fitness of BC_1_S_1_ hybrids in clay soil could be due to their mixed crop–wild genomic background and heterosis effects.

It should be noted our experiments included one location of each habitat type, albeit with large differences in conditions and replicated plots, but experiments with multiple sites for each habitat are needed to see if crop–wild hybrid individuals indeed perform better in novel habitats compared with the natural wild habitat. This pattern has been found in other species. In slender wild oat, more hybrid genotypes were able to outperform the parental lines in a greenhouse environment, representing a novel habitat, than in the original wild habitat (Johansen-Morris and Latta 2008). Similarly, radish crop–wild hybrids exhibited a higher survival rate and produced more seeds per plant relative to the wild parent in a new environment, whereas they had comparable survival rates but produced fewer seeds in the original habitat (Campbell et al. 2006). Our results also concur with those found by Hooftman et al. (2005, 2007, 2009), in crossings of the same parents as the BC lines of the current study. They found a strong heterosis effect in the clay soil averaging over all lines, but also a clear hybrid vigour breakdown over multiple generations potentially through further segregation or epistasis effects.

### Implications for crop breeding and risk assessment

The genetic processes underlying hybrid fitness have important consequences for the chances of crop (trans)gene transfer to wild populations and, therefore, for the methods of ERA. Many studies on crop–wild hybrid fitness use the average fitness of hybrid classes (Halfhill et al. 2005; Hooftman et al. 2005; Mercer et al. 2006; Campbell and Snow 2007; Huangfu et al. 2011); in case hybrid fitness is low compared with the wild parent this is taken to suggest that chances for crop allele transfer are low as well. However, our results and those of others indicate that particular hybrid genotypes may outperform the parental lines under certain environmental conditions (Burke and Arnold 2001; Johansen-Morris and Latta 2008; Hooftman et al. 2009). Furthermore, the high and significant selection differentials for fitness traits (including flowering date) and the broad-sense heritability values suggest that selection in crop–wild hybrid populations can be a dynamic and rapid process. Also, although it appears that a larger amount of crop genome decreased hybrid fitness, there was considerable spread in fitness among hybrid lines with similar crop–wild genomic ratio. Therefore, even if hybrids on average have a lower fitness, particular hybrid lines with a large amount of crop genome may exist that have a higher fitness. Thus, a lower average fitness of hybrids does not preclude gene transfer between crops and their wild relatives.

In addition, we have found that results can be cultivar-specific, that is, the fitness of hybrids depends on the specific combination of crop and wild parent and hence, fitness studies for risk assessment should include a range of wild parents (Muraya et al. 2012). Similarly, selection pressures differ across time and place, so ideally risk assessment should be performed at several locations and in multiple years (Hails and Morley 2005). ERA including hybrids of several parental lines, locations and years involves field experiments with a huge amount of time and labour. However, measuring life history traits can already lead to robust conclusions, because through QTL analysis most genomic selection patterns can be identified (Hartman et al. 2012).

### Conclusion and way forward

Our results show that there is a high likelihood in lettuce for novel crop–wild hybrids to arise that have a higher fitness than the wild parent through combinations of heterosis, linkage and transgressive segregation. This may be more likely to occur in novel habitats (Barton 2001). Consequently, this provides an avenue for introgression of crop alleles into the wild population. We did identify a genomic region on LG7 where the crop allele induced delayed flowering that was under negative selection. In this region, effects were stable across cultivars and the environments of our field experiments and it could therefore be used in transgene mitigation strategies. In such a strategy, the transgene is closely linked to a region or gene with a strong negative selection effect in the habitat of the wild type (Gressel 1999; Stewart et al. 2003).

This study is only a first step to identify the specific genes involved, and further work including the creation of Near Isogenic Lines (NILs) is being planned. Whether the detrimental effect of delayed flowering is strong enough to prevent crop (trans)gene escape will be explored further in simulation models using these empirical field data.

## Data archiving

The data used in this article are available as Supporting information.

## Supporting Information

**Data S1.** Equation 1 in Hooftman et al. 2005 no. of capitula = 50·6 (no. of branches) + 177 (no. of shoots) − 5·3 (Regression analysis: *R*^2^ = 0.51, *P* < 0·001, *n* = 315 plants).

**Table S1.** Weather conditions in Sijbekarspel (SB) and Wageningen (WG) during the period of the experiment (May—October).

**Table S2.** Positions of quantitative trait loci (QTL) for seeds produced per seed sown (SPSS) in a recombinant inbred lines population from a *Lactuca sativa* cv. Salinas × *Lactuca serriola* (UC96US23) cross using composite interval mapping.
