# Supplementary material for: Genomic and environmental selection patterns in two distinct lettuce crop–wild hybrid crosses
Source: Evol Appl. 2013 Jan 24;6(4):569–84. doi: 10.1111/eva.12043 (PMC3684739; doi:10.1111/eva.12043)
Supplement: Supplementary file 1 [file eva0006-0569-SD1.pdf]

Supporting information

Data S1 Eqn 1 in Hooftman et al. 2005

$\textit{no. of capitula} = 50\cdot6\ \textit{(no. of branches)} + 177\ \textit{(no. of shoots)} - 5\cdot3$

(Regression analysis: R^2= 0.51, P < 0·001, n = 315 plants)

Table S1 Weather conditions in Sijbekarspel (SB) and Wageningen (WG) during the period of the experiment (May—October). In Sijbekarspel, we used a data logger to record temperature and humidity levels. Daily temperature and rainfall in Wageningen was electronically available from the Haarweg weather station (www.met.wau.nl) that was located about one km from the site.

	Sijbekarspel	Wageningen
Average temperature (°C)	15.5	14.8
Relative humidity (%)	85.2	79.5
Highest average maximum daily temperature (°C)	27.4	27.9
Lowest average minimum daily temperature (°C)	5.0	4.3

Table S2 Positions of quantitative trait loci (QTL) for seeds produced per seed sown (SPSS) in a recombinant inbred lines population from a *Lactuca sativa* cv. Salinas × *Lactuca serriola* (UC96US23) cross using composite interval mapping. Other RIL QTL results are described in detail in Hartman et al. (2012). A positive additive effect indicates that crop genomic background (*L. sativa*) causes higher trait values, whereas a negative additive effect indicates that the wild genomic background (*L. serriola*) causes higher values. QTL on the same line have peak values within 5 cM. PVE = Percentage Variation Explained; Abbreviations are listed in Table 1.

LG	Trait	Position	one-LOD interval	Additive effect	PVE	LOD	Position	one-LOD interval	Additive effect	PVE	LOD
	**Sijbekarspel**						**Wageningen**				
3	SPSS						40.8	40.3–41.0	–21.57	12.5	4.8
	SPSS						75.5	72.7–77.5	21.93	13.5	4.3
5	SPSS	148.0	147.2–150.2	14.61	10.0	4.0	148.0	146.6–150.6	20.40	11.4	4.8
7	SPSS	18.5	18.4–21.5	–19.36	17.9	7.2	19.9	19.4–22.2	–28.02	21.3	8.3
	SPSS	76.7	75.1–77.3	–29.84	16.1	6.5					
